# Progress in establishing non-surgical oncology within English cancer units

**DOI:** 10.1054/bjoc.2000.1197

**Published:** 2000-07-03

**Authors:** R A Haward, Z Amir

**Affiliations:** Northern and Yorkshire Cancer Registry and Informaton Service (NYCRIS), Arthington House, Cookridge Hospital, Leeds, LS16 6QB, UK

## Abstract

In 1995 the Department of Health recommended a minimum standard of five non-surgical oncology sessions per week at Cancer Units. Postal surveys of cancer units in England were conducted in 1996 and 1999 to establish the level of provision. Substantial progress has been made from 20–60% of responding units meeting the minimum standard. © 2000 Cancer Research Campaign


British Journal of Cancer (2000) **83**(3), 284–286
© 2000 Cancer Research Campaign
doi: 10.1054/ bjoc.2000.1197, available online at http://www.idealibrary.com on IDEAL®

### Progress in establishing non-surgical oncology within English cancer units

**RA Haward and Z Amir**

Northern and Yorkshire Cancer Registry and Information Service (NYCRIS), Arthington House, Cookridge Hospital, Leeds LS16 6QB, UK

**Summary** In 1995 the Department of Health recommended a minimum standard of five non-surgical oncology sessions per week at Cancer Units. Postal surveys of cancer units in England were conducted in 1996 and 1999 to establish the level of provision. Substantial progress has been made from 20–60% of responding units meeting the minimum standard. © 2000 Cancer Research Campaign

The Department of Health (1995) report 'Policy Framework for Commissioning Cancer Services' (known as the Calman-Hine report) attached considerable importance to establishing effective multidisciplinary clinical teams. It recommended that each cancer unit should have sufficient fixed sessions from non-surgical oncologists (clinical and/or medical oncologists) to support site-specific teams for each of the common cancers. The local provision of adequate non-surgical oncology is also essential if cancer units are to deliver chemotherapy safely and appropriately.

The policy recommendation was for 'a minimum of five sessions of non-surgical oncology time, even in the smaller cancer units'. The Calman-Hine report also recommended that non-surgical oncologists visiting units should work at a cancer centre. In 1995 (the year the report was published) many District General Hospitals provided as few as one or two non-surgical oncology sessions per week, usually from a visiting clinical oncologist. Implementation of these recommendations across the country clearly required a substantial increase in unit sessions.

This paper reports the results of two questionnaire-based surveys of cancer units in England (conducted in 1996 and 1999), to establish the level of non-surgical oncology provision. The results provide an indication of progress in implementing this key policy recommendation. An outline of the results from the first questionnaire was published previously (Haward and Amir, 1997).

### Materials and methods

The survey method was a postal questionnaire to each cancer unit in England, as defined by the Calman-Hine report. Cancer centres and cancer units with radiotherapy were excluded from the survey because both have established non-surgical oncology posts for their specialized treatment roles. Their staffing levels are not specified by policy recommendations. Lists of potentially appropriate hospitals were initially acquired from the IHSM (1999). These were crosschecked with the appropriate Regional Cancer Coordinators. The first survey was conducted in March 1996 and the second in February 1999.

*Received 18 August 1999*
*Revised 11 January 2000*
*Accepted 24 February 2000*

*Correspondence to:* RA Haward

Each questionnaire was piloted and refinements made. The 1996 questionnaire was addressed to Trust Medical Directors. In 1999 we wrote to lead cancer clinicians because their role had become well-established by then and we felt they would be in closer touch with local arrangements. In this regard the questionnaire process was not strictly comparable. The two questionnaires included questions about actual sessions on given dates, but also asked about planned sessions for the coming year. The results have been pooled for analysis, and individual Trust results are not reported.

One change between the two surveys has been the trend towards Trust mergers. Some smaller cancer units have also linked up with their neighbours to form operational cancer units across more than one acute hospital site, and sometimes more than one Trust. This issue was addressed in the second questionnaire by asking specifically about the number of main acute hospitals involved. All data are for the cancer unit as a whole. A small number of responses indicated that the surveyed hospital was not part of a cancer unit, and these were excluded.

### Results

A 90% response-rate was achieved for the first questionnaire. This fell to 75% for the second questionnaire, reducing the coverage of units and raising questions as to the reasons for non-response. There were some regional differences in response rate in the first survey (75–100%), but in the second survey the range was wider (54%–91%). Table 1 shows the trends in the mean, median, and total sessions over the period. The figures quoted for 1997 and 2000 were 'planned' figures for the forthcoming year, and did not reflect actual sessions.

The proportion of surveyed units meeting the policy minimum standard has increased considerably from 20% to nearly 60%. The overall result shows an increase of two sessions in both the mean and median values. Mean sessions are consistently greater than medians reflecting a small number of units with high levels of non-surgical oncology provision. The greatest increase occurred between 1996 and 1998. However, 4 years after publication of the Calman-Hine report four out of ten cancer units who responded still do not meet the minimum standard.

Figure 1 shows the distribution of unit sessions comparing the current position with that when the Calman-Hine report was issued. This shows a significant shift to the right in the distribution

**Table 1** Number of non-surgical oncology fixed sessions per week – Cancer Units in England 1995–1999

| | Survey 1: 112 units covered (90% response rate) | | | Survey 2: 100 units covered (75% response rate) | | |
|---|---|---|---|---|---|---|
| **Operative date** | **Actual 1995** | **Actual 1996** | **Planned 1997** | **Actual 1998** | **Actual 1999** | **Planned 2000** |
| Mean sessions | 3.54 | 4.12 | 5.91 | 5.43 | 5.57 | 6.30 |
| Median sessions | 3.00 | 3.00 | 5.00 | 4.50 | 5.00 | 5.50 |
| Total sessions | 400 | 462 | 582 | 571 | 589 | 623 |
| Units with five or more sessions | 37 (20%) | 35 (31%) | 63 (56%) | 53 (53%) | 57 (57%) | 60 (60%) |

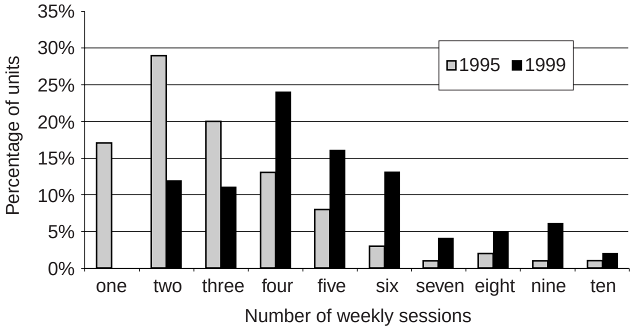

**Figure 1** Number of non-surgical fixed sessions per week in Cancer Units – April 1995 and April 1999 (in 1995 four units reported >10 sessions/week, in 1999 six units reported >10 sessions/week)

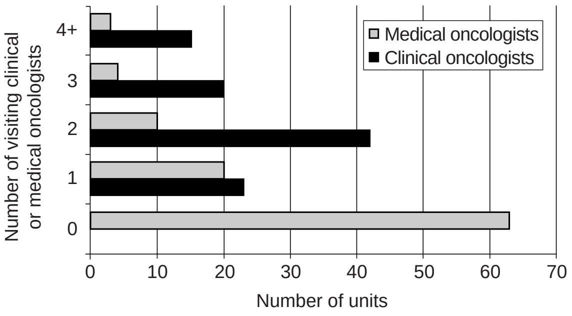

**Figure 2** Number of clinical and medical oncologists with sessional commitments in each Cancer Unit (1999 survey of 100 Units)

of unit sessions. There is a large and consistent increase in units reporting four or more sessions. No unit now reports a single session. In the second survey lead clinicians were asked to express a view as to the optimal sessions for their unit. The mean number of sessions rated as optimal was 8.5 (contrasting with a current mean of 6).

The number of medical or clinical oncologists who contributed sessions to each unit varied considerably (Figure 2). All units provided visiting sessions from clinical oncologists, the mode being involvement from two visiting oncologists. Only a third (36%) of units provided any medical oncology sessions. Units with a greater number of sessions were likely to have more oncologists involved. The relative contribution made by clinical oncologists to the provision of sessions in units has risen slightly from 77% in 1995, to 81% in 1999.

Because cancer units are a functional concept, and may involve more than one District General Hospital, the 1999 survey asked about this. Of those units responding, 68% were made up of one such hospital, 28% of two, and 4% of three. There appeared to be no relationship between the number of sessions and the number of hospitals. This may be because combined units are likely to involve smaller hospitals.

### Discussion

The policy minimum of five sessions was pragmatic rather than scientific. It was based on expert advice as to a reasonable level to enable non-surgical oncologists to contribute effectively to unit-based cancer care. While this was predominantly for the common cancers, it also provided on-site expertise to support the management or referral of less common cancers. The policy recommendation made no allowance for the size or clinical complexity of cancer units. This was because the main aim was to achieve sufficient sessions to allow oncologists to be fully involved in multidisciplinary clinical teams for breast, colorectal and lung cancer. The sessions necessary to accomplish this are largely independent of caseload.

These survey data demonstrate substantial progress in the 4 years since the publication of the policy, nearly 60% of cancer units now meet the minimum standard. However, 40% still fall short, and the lower response-rate to the second survey might conceal a preponderance of poorer performers, reducing the real level of improvement. More encouraging is the shift to the right in the distribution of unit sessions (Figure 1) with a modal value of four sessions, rather than two as in 1995. This indicates movement in the right direction. In each survey a question was asked about planned levels. While the 1997 planned levels were not achieved in 1998, they have broadly been put in place by 1999.

The original policy standard has been a valuable target and provided a benchmark for measuring progress. It should be revised in the light of the experience of implementing modern multidisciplinary care in a cancer unit setting, and of the associated devolution of adjuvant chemotherapy for common cancers. Revision should also reflect the range of clinical workloads. The wide variation in sessional provision shown in this survey, together with the opinions of lead clinicians that the optimum has a mean of 8.5 sessions, suggests that further refinement of the standard is required.

Clinical oncologists remain the dominant discipline in unit non-surgical oncology, increasing their proportion of sessions over the

*© 2000 Cancer Research Campaign*

last 4 years. Medical oncology sessions are now provided in about a third of all units. There are clearly different staffing models operating in units, as a third now have three or more clinical oncologists providing their sessions. This may represent some site-specialization among the visiting oncologists.

Substantial progress has been made in the majority of units towards achieving the minimum standard. In about 30% of units the level of provision is higher, sometimes considerably higher, than the minimum recommended. Of concern is the fact that about a quarter of units have failed to increase their non-surgical oncology sessions above three per week. The lower response rate in the 1999 survey raises the question as to how far non-responders might be different from the observed picture.

### REFERENCES:

Department of Health (1995) *Policy Framework for Commissioning Cancer Services: a report by the Expert Advisory Group on Cancer to the Chief Medical Officers of England and Wales*. Her Majesty's Stationary Office: London

Haward RA and Amir Z (1997) Cancer units are implementing changes from generic to specialist practice (letter). *BMJ* **314**: 677

IHSM (1999) *Health and Social Services Year Book 1999/2000*. FT Business-Directories: London, ISBN 1–184083–132

© 2000 Cancer Research Campaign
